# Supplementary material for: Molecular Lipopolysaccharide Di-Vaccine Protects from Shiga-Toxin Producing Epidemic Strains of Escherichia coli O157:H7 and O104:H4
Source: Vaccines (Basel). 2022 Nov 1;10(11):1854. doi: 10.3390/vaccines10111854 (PMC9692684; doi:10.3390/vaccines10111854)
Supplement: Supplementary file 1 [file vaccines-10-01854-s001.zip › vaccines-1974051-supplementary.pdf]

Supplement

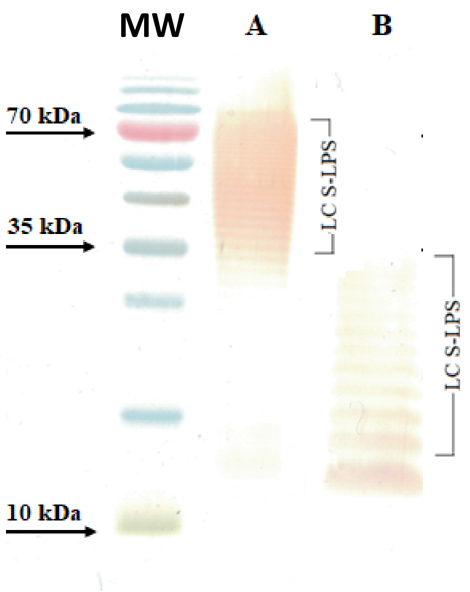

Figure S1. Uncropped image of silver-stained SDS-12% polyacrylamide gel electrophoresis of S-LPS from *E. coli* O157 (A) with molecular weight of 35-70 kDa and *E. coli* O104 (B) with molecular weight of 15-35 kDa. LC S-LPS indicate S-LPS with long-chain O-polysaccharide. MW – molecular mass standard. Due to the size difference sample (B) is running out of the gel before bands in sample (A) get fully resolved.

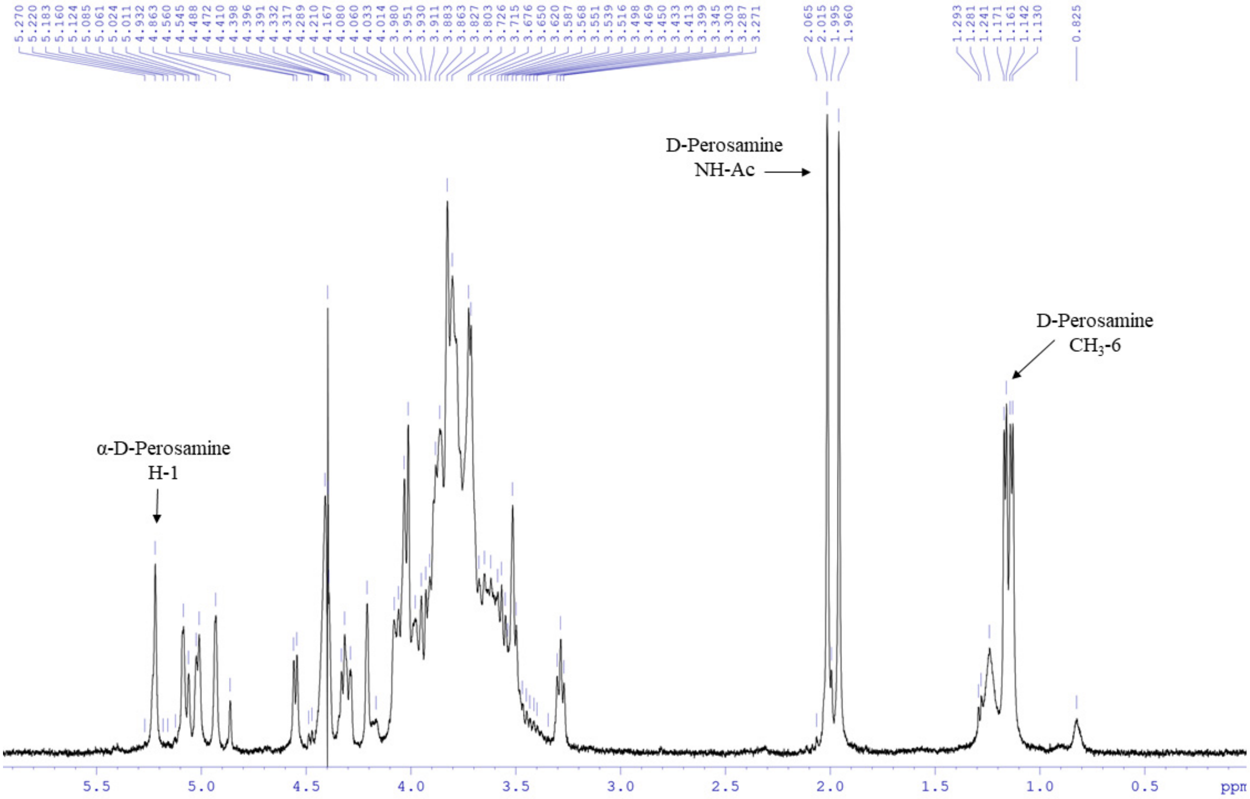

Figure S2. ^{1}H-NMR spectrum (500MHz, 50ºC) of the O-chain of the 3-O-Acyl-LPS from *E. coli* O157. The chemical shift assignments for α-D-Perosamine (α-D-PerNAc) were made by the comparison with literature data [1].

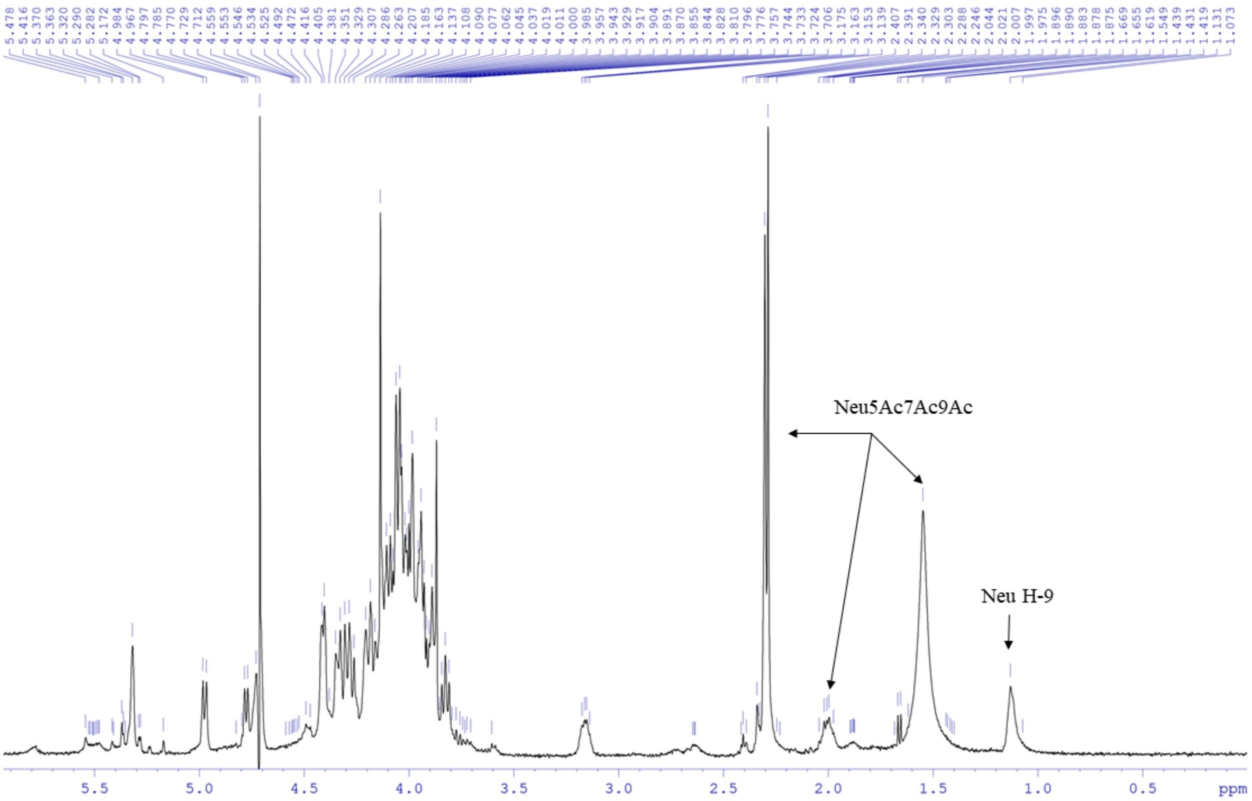

Figure S3. ^{1}H-NMR spectrum (500MHz, 50ºC) of the O-chain of the 3-O-Acyl-LPS from *E. coli* O104. The chemical shift assignments for N-acetyl-7,9-di-O-acetylneuraminic acid (α-Neu5Ac7Ac9Ac) were made by the comparison with literature data [2].

Supplemental references

[1] Perry, M.B. "Structure of the O-chain polysaccharide of the phenol-phase soluble lipopolysaccharide of Escherichia coli 0:157:H7". *Biochemistry and Cell Biology*. **1986**, *64(1)*, 21-28

[2] Gamian, A. "The structure of the sialic acid-containing Escherichia coli O104 O-specific polysaccharide and its linkage to the core region in lipopolysaccharide". *Carbohydrate Research*. **1992**, *236*, 195-208
